# The Role of Fathers in Promoting Exclusive Breastfeeding

**DOI:** 10.7759/cureus.30363

**Published:** 2022-10-16

**Authors:** Jayesh Agrawal, Swarupa Chakole, Chetna Sachdev

**Affiliations:** 1 Community Medicine, Jawaharlal Nehru Medical College, Datta Meghe Institute of Medical Sciences, Wardha, IND

**Keywords:** positive parenting, breast-feeding, mother and child health, father's role, child nutrition, father, exclusive breast feeding

## Abstract

The cornerstone of a newborn's nutrition is breastfeeding. Due to its well-known benefits for mothers, children, and society in the short and long term, the World Health Organization (WHO) and United Nations Children's Fund (UNICEF) recommend it as the best way of feeding the baby during the first six months of life. The family, particularly the spouse, may significantly influence the baby's quality of nursing. On a global scale, previous studies have demonstrated that family members (such as a mother's spouse, partner, or grandmother) not only affect her choice to start and endure breastfeeding but also significantly contribute to the early postnatal period's cessation of appropriate breastfeeding. A father's knowledge and attitude are fundamental in this regard, as he has the most critical role in helping women with parenting and feeding their babies.

Furthermore, because the father's role is considered important in a family, the partner's or wife's perception of the father's attitude may alter her subjective criteria about exclusive breastfeeding (EBF). Future initiatives should target new mothers and their spouses to see how they may provide the most beneficial assistance to new mothers. The main focus should be on targeting newly married couples. The father's lack of involvement may be due to the hierarchical structure of power within households. Because of this hierarchical aspect, partners or fathers may significantly influence a mother's choice to nurse their child correctly. Household chores, childcare for grown-up children, guaranteeing the well-being of their spouse, preparing meals, recognizing a newborn's hunger cues, burping, and changing the infant's diaper after feedings, all these factors indirectly support the mother in initiating and enduring the nursing of a child.

## Introduction and background

Breastfeeding is the foundation of a newborn’s nutrition. For the first six months of a baby's life, two organizations, the World Health Organization (WHO) and the United Nations Children's Fund (UNICEF), suggest it as the exclusive method of feeding the newborn due to its well-known advantages for the mother, child, and society in the short and long term [1]. However, outside of the confines of a medical setting, the presence of social support is a significant factor in determining the results of breastfeeding. An infant breastfed for their whole first year of life may be less likely to suffer from allergies, obesity, constipation, diarrhea, and other illnesses later in childhood [2]. In addition, there is evidence that breastfeeding can enhance a child's cognitive and motor development and reduce the risk of sudden infant death syndrome (SIDS) [3].

Early initiation of breastfeeding (EIBF) and exclusive breastfeeding (EBF) are excellent ways of increasing the number of children who live to adulthood worldwide. EIBF is giving human breastmilk to babies within the initial 24 hours of birth. In contrast, EBF means feeding babies between the ages of 0-five months only human breastmilk (and minerals, oral rehydration solution, or drops/syrups of vitamins or medicines as needed) without providing any other nutrition or water [4]. Thus, breastfeeding needs to be initiated and promoted, and awareness should be created. The family members, mainly the husband, may play a central role in the quality of breastfeeding the baby receives. Previous research has shown, on a global scale, that family members (such as a mother's husband, spouse, or grandparents) do not only single-handedly impact a mother's wish to begin and endure breastfeeding but also play a noteworthy part in the early termination of appropriate breastfeeding in the initial period after delivery. Family members impact a mother's choice to induct and endure breastfeeding [4]. Some other systematic review articles also suggested that breastfeeding interventions that involved updating the partner or father's knowledge or educating him about it resulted in an improvement in breastfeeding outcomes.

## Review

Search strategy

A narrative study or review was carried out using the PubMed archive, a platform that can be used to conduct reviews of many original articles and to find information on breastfeeding interferences that discloses breastfeeding barricades and promoters available in the English language. There were no topographical boundaries, but only studies in English were comprised because of the source boundaries. The research was initiated using keywords and combinations such as "breastfeed" and "father" or "partner" or "spouse" and "role" or "promotion" or "influence" or "support".

The criteria which were included and excluded in the review are presented in Table 1.

**Table 1:** Inclusion and exclusion criteria

Inclusion criteria	Exclusion criteria
Availability of complete text in the English language	Cost-effectiveness studies
Assess the efficacy of interference on breastfeeding outcomes	Measures indirect indicators of breastfeeding

Mother’s knowledge and EBF experience

Individuals have the potential to achieve greater levels of performance in practice if they possess the appropriate contextual information, but having insufficient knowledge unquestionably results in low performance. When it comes to the practice of exclusive breastfeeding, the attitude of the mother is quite important, and it is mainly characterized by maternal knowledge [5]. The lack of a favorable attitude towards EBF and a lack of sufficient information may result in a decreased likelihood of initiating and maintaining EBF at the appropriate times [6]. In addition, a person's level of education, coupled with their prior experiences, may both be significant sources of information and help shape an attitude about their attitude to breastfeeding.

Father’s knowledge and support in EBF

The researchers focused on mothers' knowledge of breastfeeding practices. Some researchers have also studied the father's command, attitude to support, and participation in breastfeeding practices. Although this task of nourishing the child is primarily taken care of by the mothers, the male partners, on the other hand, can increase the nourishment exponentially through their knowledge and support of EBF practices [7]. Fathers can increase breastfeeding potential because a father who is adequately educated can support his wife or partner and help her learn more about starting and continuing breastfeeding. Men have the most significant role to play in assisting mothers with parenting and feeding their infants, and their knowledge and attitude are crucial in this respect. Furthermore, because the father's role is regarded as the most important in a family, the partner's or wife's observation of the father's mentality has the potential to change her subjective standards on EBF [8]. 

Role of the father in supporting EBF

A father's main responsibility is to become a supporter and enabler of breastfeeding the infant [9]. Housework, taking care of older children, caring for their partner's well-being, meal preparation, recognizing an infant's hunger cues, burping the baby, and changing the infant's diaper after feeding should all be part of the responsibilities taken up by the father[10].

**Figure 1:**
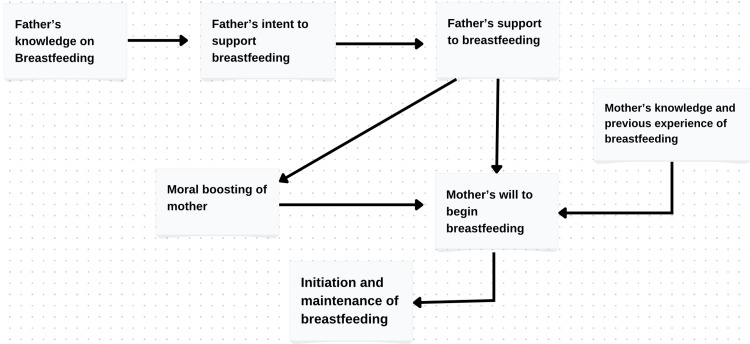
Role of fathers in promoting breastfeeding [3]

The father's knowledge of breastfeeding causes him to want to support it, and his encouragement of breastfeeding enhances the mother's confidence to start it. A mother's earlier experiences and knowledge also encourage her to start and continue breastfeeding her children (Figure 1).

The effect of the father’s support in EBF

Appropriate breastfeeding support from a partner is vital for the baby's nutrition and also impacts a new mother's decision to start, endure, or discontinue nursing in the initial period after delivery. Although partner nursing assistance can take many different forms, the effects on the start of breastfeeding, duration, and selectivity are almost always positive, especially when verbal support is provided [11]. Partner responsiveness, assistance in avoiding and dealing with breastfeeding issues, and assistance with household work and childcare tasks are all important and critical types of partner-supporting activities that result in optimal breastfeeding behavior [12]. Even though various analyses and studies suggested that specific types of husband support might have a significant impact on breastfeeding success in society, they also showed that there is still a dearth of high-quality, demographic-based research on the impact of partners' specific roles in breastfeeding behaviors. In the studies, the fathers also experienced the urge to be emotionally and practically there to support the mother during breastfeeding. They could support the mother through love, affection, and motivation, and by encouraging her that breastfeeding is a team sport [13]. They may perceive the opportunity to nourish their child as a source of constructive knowledge and as a contributive element to the creation of the father and infant bonding over either the feeding of the expressed breast milk by bottle or infant formula throughout the breastfeeding time or giving solids whenever the child is prepared [14,15].

Support interferences for fathers during breastfeeding

Researchers that looked into the connection between the quality of life (QOL) of fathers and the feeding habits of their babies discovered that fathers of breast-nourished babies had higher QOL ratings than fathers of bottle-nourished babies [16]. This was seen because breastfed babies were found to be healthier than bottle-fed ones in their early childhood. They should be made aware of the various ways they can assist with breastfeeding and how beneficial all forms of support provided by them can be. A large portion of the fathers in the studies required online supervision from father-to-father mentoring networks, prenatal support groups, breastfeeding literature, health care professionals, and other sources of technology [17]. Concerning the support provided by healthcare professionals, some of them reported feeling overburdened with information that was occasionally conflicting [18]. In contrast, others voiced unhappiness with the lack of programs and resources that were specifically created for them [19]. The majority claimed that information may not have reached them at all or may have been delivered to them via their partner or family members in the form of pamphlets. A few of them mentioned getting information from healthcare professionals directly [20]. On the other hand, several men who received prenatal advice from healthcare professionals thought the material was beneficial [21]. There were mixed emotions regarding the interventions created for them. Supporting the mother in the post-natal period, especially during the period of EBF, represents the fathering and parenting role of a man. Thus, their insight that their care results in a positive breastfeeding experience for the mother pushes them to better parenting. Each partner has a vital role to play and must contribute in some way, but both must be willing to stand up when necessary. Additionally, they must synchronize their efforts and have faith in one another to complete their necessary responsibilities. This needs constant contact, observation, and focus on what the other person is performing. Then, if any one partner requires assistance, the other can be there for them and can move aside if everything is under control.

Discussion

Breastfeeding is a natural and inalienable right for both mothers and their children. Every effort should be made to encourage, practice, and continue exclusive breastfeeding for the first six months of a child's life [22]. The World Health Organization (WHO) endorses that mothers nurse their babies entirely for the duration of the first six months of their babies' lives due to the significant role that breastfeeding plays in a child's development and growth, as well as the positive effects that EBF has on the health of the mother. After that, continued breastfeeding should be practiced with the addition of other foods and supplements until the child reaches the age of two [23]. The WHO and the United Nations International Children's Emergency Fund (UNICEF) collaborated in 1992 to develop the "Ten Steps to Successful Breastfeeding," which went on to become the foundation of the Baby Friendly Hospital Initiative (BFHI). The Baby-Friendly Community Initiative (BFCI) is also an extension and unification of the BFHI. One requires seven ladders to meet global standards, among which one suggests expanding breastfeeding education to the whole family, and one offers a warm and friendly environment for breastfeeding families [24].

The choices that women make about nursing are significantly influenced by the opinions of their husbands. Initiation and duration of breastfeeding are both influenced by the emotional and physical assistance that fathers provide. At the same time, this support is a morale booster for mothers, who acquire a better sense of self-efficacy if they feel empowered by their partners [25]. In cross-sectional studies, it was found that women who had partners who were either apathetic or opposed to nursing had lower levels of confidence in their capacity to breastfeed than did women who had partners who were supportive of breastfeeding on both a passive and active level. The support of a partner is a significant predictor of a mother's confidence in her ability to breastfeed, which is truly independent of the mother's level of prior nursing experience or the age of the newborn. There is an ever-increasing body of research that lends credence to the notion that fathers should be included in the policymaking process regarding nursing and also in the process of acquiring supportive behaviors that are both positive and functional after childbirth. This research backs up the idea that fathers need to be included in the decision-making process regarding nursing. It will be necessary to deliver material to fathers on the most efficient means by which they can support their partners in overcoming challenges related to breastfeeding a child [26].

Families may manage these challenges and support breastfeeding positively by being ready and having truthful expectations about how it may affect them as individuals, as couples, or as households [27]. The breastfeeding work intended for fathers must contain constructive representations of male parents as members of the nursing team. Any prenatal material for dads would also benefit from using the term "father" rather than "parent," which men may erroneously confuse with "mother" [28]. To make education more accessible to working men, mobile prenatal services where healthcare professionals visit the offices of expectant or new fathers and deliver instruction during break times can also be considered a good choice [29]. In antenatal sessions, mentors can be used to normalize their emotional state and understanding and guarantee them that their connection with the infant won’t be jeopardized in the long term by breastfeeding [30].

The research was carried out, the critical components of which were interviews and group discussions with mothers, and an online questionnaire was produced for the fathers. It was shown that breastfeeding continued for a more extended period when the partners uttered words of encouragement to mothers, such as "do your best," acknowledged them for the effort they put into nursing, and provided them with emotional support [31]. According to the findings of the study, improved breastfeeding practice was also associated with partners who anticipated the requirements of new mothers and were successful in meeting those demands, as well as partners who displayed a dedication to breastfeeding [32]. In addition, the research also found that improved breastfeeding practice was associated with an increased number of partners who breastfed their children. The influence of companion support on breastfeeding start, duration, and exclusivity was generally good, even if there were differences in the forms of companion breastfeeding support. This was especially true when the support was given in the form of spoken inspiration. The reaction of the partner, help in evading and handling breastfeeding issues, and help with home and child care tasks are all significant and crucial sorts of partner-supporting activities that lead to excellent breastfeeding behavior. Even though the study demonstrated that certain kinds of support from a partner could have a significant influence on the level of success that breastfeeding has in a community, it also demonstrated that there is still a scarcity of good quality, population-based education on the effect of the precise role that fathers play in breastfeeding practices [4].

All this shows that in the future, families should focus their efforts on new mothers and support them in various ways. The primary goal of these efforts should be to involve fathers in child nutrition [20]. Less involvement of fathers in the early nourishment of children is a result of the fact that, given the hierarchical structure of power within families, partners could have a significant impact on a mother's choice to properly breastfeed their child [33]. In addition, including grandmothers in upcoming breastfeeding activities (if they can participate) would help maximize the benefits since, according to previous evaluations, grandmothers may also impact the nursing behavior of society. If they can participate, this will help [33].

Limitations

There are some limitations to the study that should be emphasized. The article does not address circumstances when there are complications with the breastfeeding process. The maternal complications could be sore nipples, reduced breastmilk production, or mastitis, and the complications associated with the baby could be the inability to latch on to the breasts. Moreover, the review included studies in the English language only, which is why it could not contain the whole spectrum of information. The use of unpublished works also minimized the sources for reference.

## Conclusions

The review demonstrated that a mother's choice to commence and sustain breastfeeding in the initial postnatal period could be influenced by specific and suitable assistance by the partner. The most common type of assistance for new mothers to enhance their nursing routines was verbal encouragement from their partners. Additional specific types of support activities that led to better breastfeeding practices included assistance with household and child care responsibilities; prevention and management of breastfeeding difficulties; and being sensitive and considerate to the nursing mother's needs from the husband. Treatments for nursing new mothers should reflect partners' involvement and their duties to maximize outcomes. Supporting fathers in their various nursing responsibilities and recognizing them as important members of the breastfeeding triad can also help men overcome feelings of inadequacy and loneliness, boosting their well-being and resulting in several significant benefits for the entire family. For breastfeeding to be effective, the father's practical and emotional assistance is a crucial component. This boosts the mother's self-esteem and enables her to maintain a sufficient milk supply. While nursing is still exclusively a woman's responsibility in society, the absence of a partner's crucial assistance might be a missed opportunity for better parenting as a father.
